# Blood Urea Nitrogen to Creatinine ratio in Differentiation of Upper and Lower Gastrointestinal Bleedings; a Diagnostic Accuracy Study

**Published:** 2019-06-02

**Authors:** Seyyed Mahdi Zia Ziabari, Siamak Rimaz, Afshin Shafaghi, Maryam Shakiba, Zahra Pourkazemi, Elnaz Karimzadeh, Melika Amoukhteh

**Affiliations:** 1 Department of Emergency Medicine, School of Medicine, Guilan University of Medical Sciences, Rasht, Iran.; 2 Anesthesiology Department, Anesthesiology Research Center, Alzahra Hospital, Guilan University of Medical Sciences, Rasht, Iran.; 3 GI Cancer Screening and Prevention Research Center, School of Medicine, Guilan University of Medical Sciences, Rasht, Iran.; 4 School of Health, Guilan University of medical sciences, Rasht, Iran.; 5 Road Trauma Research Center, School of Medicine, Guilan University of Medical Sciences, Rasht, Iran.; 6 Poursina Clinical Research Development Unit, School of Medicine, Guilan University of Medical Sciences, Rasht, Iran.; 7 School of Medicine, Guilan University of Medical Sciences, Rasht, Iran.

**Keywords:** Gastrointestinal hemorrhage, blood urea nitrogen, creatinine, clinical decision-making, decision support techniques

## Abstract

**Introduction:**

Finding easily accessible and non-invasive methods for differentiating various sources of gastrointestinal (GI) bleeding before performing endoscopy and colonoscopy is of great interest. The present study was designed with the aim of evaluating the screening performance characteristics of blood urea nitrogen (BUN) to Creatinine (Cr) ratio in this regard.

**Methods:**

The present diagnostic accuracy study was performed on patients with acute GI bleeding presenting to emergency department from 2011 to 2016, in a retrospective manner. BUN/Cr ratio was calculated for all patients and its accuracy in differentiation of upper and lower GI bleedings, confirmed via endoscopy or colonoscopy, was evaluated.

**Results:**

A total of 621 patients with the mean age of 59.49±17.94 (5 – 93) years were studied (60.5% male). Area under the receiver operating characteristic (ROC) curve of BUN/Cr ratio for predicting the source of GI bleeding was 0.63 (95% CI: 0.57 – 0.68). Sensitivity, specificity, positive and negative predictive values, and positive and negative likelihood ratios of BUN/Cr ratio at 35 cut-off point were 19.63% (95%CI: 16.69 – 23.45), 90.16% (95%CI: 83.11 – 94.88), 89.09 (95%CI: 81.35 – 93.98), 21.53 (95%CI: 18.09 – 25.39), 8.16 (95%CI:4.76 – 13.98), and 3.65 (95%CI: 3.44 – 3.87), respectively.

**Conclusion:**

Considering the relatively proper specificity and positive predictive value of BUN/Cr ratio, in cases that bleeding source cannot be determined using other non-invasive methods, values higher than 35 can predict upper GI bleeding with high probability. However, due to the low sensitivity, values less than 35 are not diagnostic.

## Introduction:

Gastrointestinal (GI) bleedings are among common causes of emergency department visits and there are about 800000 visits with complaint of GI bleeding every year only in the United States, about half of which will need hospitalization (1). GI bleedings are divided into two groups of upper (above treitz ligament) and lower (below treitz ligament) based on the site of bleeding. Annual incidence of upper GI bleeding is higher than lower GI bleeding and mortality due to upper GI bleeding has been estimated to be up to 40% for those with unstable hemodynamics (2, 3). Meanwhile, this rate has been between 10% and 20% for lower GI bleeding (4).

Differentiation of upper and lower GI bleeding is very important for choosing the proper treatment modality. For this purpose, various tools such as history taking, clinical examination, and laboratory parameters (like hemoglobin, platelet, C reactive protein) as well as invasive diagnostic treatment methods such as endoscopy and colonoscopy are available (5-7).

Finding easily accessible and non-invasive methods for differentiating various types of upper GI bleeding before performing endoscopy and colonoscopy is of great interest. Among the laboratory parameters that can be used for differentiation of upper and lower GI bleeding, blood urea nitrogen (BUN) can be pointed out (8). Decrease in blood flow to the kidney, secondary to losing volume due to bleeding and also digestion of blood in the digestive system and metabolization of proteins resulting from it to BUN in the urea cycle are introduced as the reasons for increase in the level of this biomarker (3, 8). If the cause of azotemia is degradation of blood in the digestive system, it is expected that higher BUN levels more strongly correlate with upper GI bleeding.

Some studies have used BUN to creatinine (Cr) ratio as an index to differentiate upper and lower GI bleedings and have shown that higher BUN to Cr ratio is associated with higher probability of upper GI bleeding (8, 9). On the other hand, other studies have shown that Bun and BUN/Cr ratio levels lack the required accuracy in differentiation of upper and lower GI bleeding (10, 11). Therefore, considering the existing disagreements, the present study was designed and performed with the aim of evaluating the screening performance characteristics of BUN to Cr ratio in differentiation of upper and lower GI bleedings.

## Methods:

Study design and setting

The present diagnostic accuracy study was performed on patients with acute GI bleeding presenting to the emergency department of Razi Hospital, Rasht, Iran, from 2011 to 2016 and screening performance characteristics of BUN to creatinine ratio regarding source of bleeding (upper or lower digestive system) were evaluated. Protocol of the study was approved by the ethics committee of Guilan University of Medical Sciences under the number IR.GUMS.REC.1396.432 and researchers adhered to confidentiality of patients’ data. This study was carried out in a retrospective manner using patients’ medical profiles.

***Participants***

All the patients that had presented to the emergency department with acute GI bleeding manifesting as hematemesis, melena, and hematochezia and less than 24 hours had passed from their bleeding were included in the study. These patients should have had BUN and Cr evaluations on admission and finally, their source of GI bleeding should have been determined using a reliable method such as endoscopy or colonoscopy with evidence present in the profile. 

Patients with a history of confirmed renal failure (where Cr levels were above 120 µmol/L or 2.16 mg/dL in the last 3 months or they had undergone dialysis), those who had received blood transfusion during the 24 hours prior to admission, and those showing evidence of thrombocytopenia or coagulopathy or having a history of injecting cephalosporins or any other drug interfering with BUN or Cr evaluation, as well as patients with both upper and lower GI bleeding were excluded from the study. 

***Data gathering***

Census sampling was used for data gathering. A checklist consisting of demographic data (age and sex), underlying illnesses (diabetes, hypertension, cardiovascular disease, liver cirrhosis), history of GI bleeding, history of upper or lower GI cancer, history of cigarette, alcohol, or tobacco addiction, history of medication use (Aspirin, non-steroidal anti-inflammatory drugs (NSAID), steroid, warfarin, Iron or Bismuth), and laboratory findings (Cr, BUN, platelet (Plt), hemoglobin (Hb)) as well as the accurate source of bleeding according to endoscopy or colonoscopy findings was filled for all the patients presenting to the emergency department with GI bleeding during the mentioned time by referring to their medical profile. 

Patients whose source of bleeding was determined to be over the treitz ligament using endoscopy were reported as upper GI bleeding, and if the source of bleeding was not detected in endoscopy and the site of bleeding was not seen in colonoscopy, the case was considered as a lower GI bleeding with negative colonoscopy and if the source of bleeding was not detected in endoscopy or endoscopy was not performed and bleeding was observed in colonoscopy, the case was considered as lower GI bleeding with positive colonoscopy. Two medical interns were responsible for gathering data under supervision of an emergency medicine specialist.

***Statistical analysis***

Minimum required sample size was determined to be 630 patients based on 69% sensitivity for BUN/Cr ratio (9) and estimating and considering 20% prevalence for upper GI bleeding and type 1 error of 0.05 and 8% desired precision. All the data were analyzed in STATA version 13.0 (StataCorp, College Station, TX, USA) statistical software after gathering. To compare the groups regarding qualitative indices, chi square, and for comparing quantitative indices, t-test were used. Sensitivity, specificity, positive and negative predictive values and positive and negative likelihood ratios were calculated and reported for the best cut-off point of BUN/Cr ratio in differentiation of upper and lower GI bleedings. The best cut-off point was calculated using the area under the receiver operating characteristic (ROC) curve. P values ≤ 0.05 were considered significant.

## Results:

**Figure 1:**
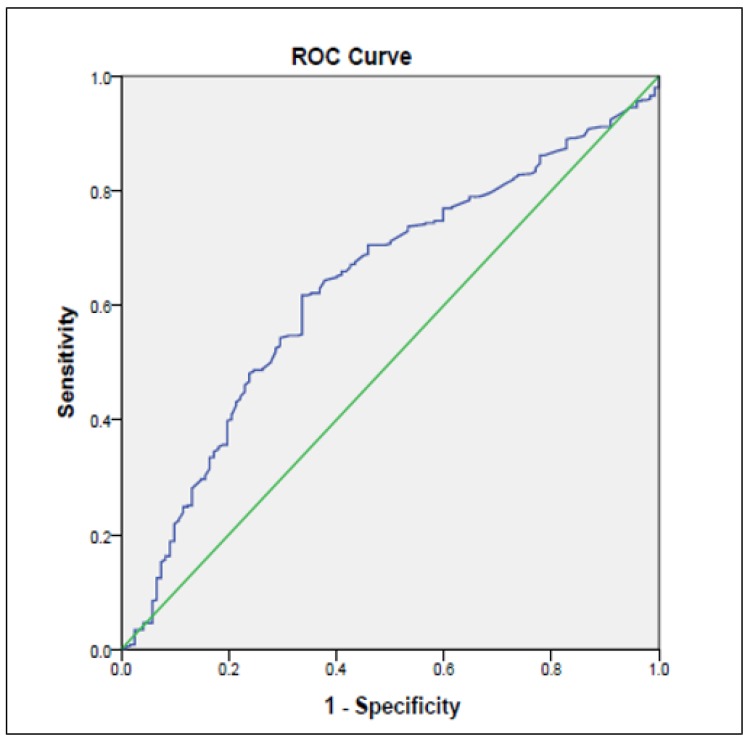
Area under the receiver operating characteristic (ROC) curve of blood urea nitrogen (BUN)/Creatinine ratio for predicting the source of gastrointestinal bleeding (upper or lower)

A total of 621 patients with the mean age of 59.49±17.94 (5 – 93) years were studied (60.5% male). Based on the results of endoscopy and colonoscopy, 499 (80.35%) bleeding cases were related to the upper digestive system and 122 (19.65%) cases were related to the lower digestive system. Tables 1 and 2 have compared the baseline and laboratory characteristics of patients with upper and lower GI bleeding. The two groups were similar regarding sex distribution (p = 0.72), history of underlying illnesses (p > 0.05), history of drug abuse (p > 0.05), and history of taking medications (p > 0.05). Mean age of the patients with lower GI bleeding was about four years lower (p = 0.028), BUN/Cr ratio was significantly higher in those with upper GI bleeding (25.90 ± 15.16 versus 21.16 ± 13.77; p = 0.001). Area under the ROC curve of BUN/Cr ratio for predicting the source of GI bleeding was 0.63 (95% CI: 0.57 – 0.68) (figure 1). The best cut-off point of BUN/Cr ratio for predicting the source of bleeding was estimated as 35.13. Sensitivity, specificity, positive and negative predictive values, and positive and negative likelihood ratios of BUN/Cr ratio at this cut-off point were 19.63% (95%CI: 16.69 – 23.45), 90.16% (95%CI: 83.11 – 94.88), 89.09 (95%CI: 81.35 – 93.98), 21.53 (95%CI: 18.09 – 25.39), 8.16 (95%CI:4.76 – 13.98), and 3.65 (95%CI: 3.44 – 3.87), respectively. 

## Discussion:

Based on the results of the present study, considering the acceptable specificity and positive predictive value of BUN/Cr ratio, in cases that the source of bleeding cannot be determined via other non-invasive methods, values higher than 35 predict an upper source for the GI bleeding with high probability.

In this study, the rate of BUN is higher in patients with upper GI bleeding compared to those with lower GI bleeding. The level of BUN in blood increases following digestion of a high volume of blood or protein in the digestive system. The source of voluminous GI bleeding is mostly above the treitz ligament and therefore, blood has more time for absorption and catabolism in the digestive system. Therefore, it is expected that upped GI bleedings have higher BUN compared to lower GI bleedings. On the other hand, the cause of lower GI bleedings is usually in the colon and considering the low absorption of nutrients in colon, it is expected that patients with lower GI bleedings have lower BUN levels. Meanwhile, upper GI bleeding cases probably have higher creatinine levels due to losing more volume.

In the present study, mean BUN/Cr ratio was 25.90 ± 15.16 in patients with upper GI bleeding and 21.16 ± 13.77 in those with lower GI bleeding (p value = 0.001).

In a study in 2015, which was performed on 141 patients with upper and lower GI bleeding, Tomizawa et al. showed that BUN measure alone can differentiate upper and lower GI bleedings. In their study, they considered BUN>21 as the threshold and expressed that BUN over 21 indicates upper GI bleeding with 36.4% sensitivity and 93% specificity (7).

In a study conducted on 124 patients with GI bleeding, Ernest et al. showed that BUN/Cr ratio significantly correlated with upper GI bleeding (8).

Additionally, in the study by Urashima et al. on 85 children with GI bleeding, BUN/Cr ratio was significantly different between the upper and lower GI bleeding groups. BUN/Cr ratio of 30 or higher had 98% specificity and 68.8% sensitivity in detection of upper GI bleeding (9). 

Richards et al. also conducted a retrospective study on 74 patients with upper GI bleeding and 52 patients with lower GI bleeding and showed that none of the patients with lower GI bleeding had a BUN/Cr ratio equal to or higher than 36, while 38% of the patients with upper GI bleeding had a BUN/Cr ratio equal to or higher than 36 (10).

As can be seen, various studies have been in line with our study regarding significance of BUN/Cr ratio in differentiation of the source of bleeding.

**Table 1:** Comparing baseline characteristics of patients with upper and lower gastrointestinal (GI) bleedings

**Variable **	**Source of GI bleeding**		**P value**
	**Upper**	**Lower**	
**Sex **			
Male	298 (59.7)	78 (63.9)	0.72
Female	201 (40.3)	44 (36.1)	
**Age (year) **			
Mean ± standard deviation	60.27 ± 17.91	56.29 ± 17.76	0.028
**Underlying illness**			
Diabetes	110 (22.0)	24 (19.7)	0.32
Hypertension	195 (39.1)	45 (36.9)	0.19
Cardiovascular diseases	91 (18.2)	19 (15.6)	0.47
Cirrhosis	17 (3.4)	0 (0.0)	0.039
History of GI bleeding	97 (19.4)	28 (23)	0.38
History of GI cancer	8 (1.6)	5 (4.1)	0.08
**History of addiction**			
Alcohol	22 (4.4)	4 (3.3)	0.5
Cigarette	104 (20.8)	23 (18.9)	0.6
Drugs	76 (15.2)	22 (18)	0.44
**History of Medication**			
NSAID	94 (18.8)	23 (18.9)	0.99
Corticosteroid	6 (1.2)	1 (0.8)	0.72
Warfarin	22 (4.4)	8 (6.6)	0.32
Iron	62 (12.4)	19 (15.6)	0.35
Bismuth	6 (1.2)	2 (1.6)	0.70

Data are presented as frequency (%). NSAID: Non-Steroidal Anti-Inflammatory Drugs.

**Table 2:** Comparing the laboratory findings of patients with upper and lower gastrointestinal (GI) bleedings

**Variable **	**Source of GI bleeding**		**P value**
	**Upper**	**Lower**	
Hemoglobin (mg/dL)	9.36 ± 2.45	9.90 ± 2.42	0.014
BUN (mg/dL)	28.81 ± 18.62	20.62 ± 14.14	0.001
Creatinine (mg/dL)	1.25 ± 1.15	1.00 ± 0.33	0.032
Platelet (mg/dL)	21970 ± 89.90	238065 ± 81.92	0.007
BUN/Creatinine ratio	25.90 ± 15.16	21.16 ± 13.77	0.001

Data are shown as mean ± standard deviation. BUN: blood urea nitrogen.

It seems that in cases with acute GI bleeding and when patients are hemodynamically stable, if the diagnosis of bleeding source is ambiguous based on the clinical examination of the patient, and aspiration of nasogastric discharge of the patient does not help, BUN/Cr ratio can be applied as an index for differentiating the site of bleeding in the digestive system. For this purpose, using a simple and inexpensive blood test, if this ratio was calculated to be over 35 (considering the positive predictive value and high specificity) the source of bleeding can be considered upper digestive system with high probability and if the ratio was lower than 35 (considering the low sensitivity and negative predictive value) this index does not help in differentiating the source of bleeding and other diagnostic measures are needed.

***Limitations:***

Hypovolemia is a major factor in elevating plasma urea levels, and it would have been better if patients were matched regarding hypovolemia rate using hematocrit and urine output rate.

## Conclusion:

Considering the relatively proper specificity and positive predictive value of BUN/Cr ratio, in cases that bleeding source cannot be determined using other non-invasive methods, values higher than 35 can predict upper GI bleeding with high probability. 
